# Correlation Between Serum Uric Acid and Body Fat Distribution in Patients With Polycystic Ovary Syndrome

**DOI:** 10.3389/fendo.2021.782808

**Published:** 2022-01-25

**Authors:** Yuqin Zhang, Meili Cai, Diliqingna Dilimulati, Ziwei Lin, Hang Sun, Ran Cui, Hongxiang Fei, Xinxin Gao, Qiongjing Zeng, Xiaowen Shao, Manna Zhang, Shen Qu

**Affiliations:** ^1^ Department of Endocrinology and Metabolism, Shanghai Tenth People’s Hospital, Tongji University School of Medicine, Shanghai, China; ^2^ National Metabolic Management Center, Shanghai Tenth People’s Hospital, Shanghai, China; ^3^ Department of Traditional Chinese Medicine, Shanghai Tenth People’s Hospital, Tongji University School of Medicine, Shanghai, China; ^4^ Department of Obstetrics and Gynecology, Shanghai Tenth People’s Hospital, Tongji University School of Medicine, Shanghai, China

**Keywords:** polycystic ovary syndrome (PCOS), serum uric acid (SUA), hyperuricemia, body fat distribution, visceral adipose tissue (VAT)

## Abstract

**Objective:**

This study aims to investigate the correlation between serum uric acid levels and body fat distribution in patients with polycystic ovary syndrome (PCOS).

**Methods:**

Between May 2017 and March 2021, a total of 199 patients with PCOS were recruited from the Department of Endocrinology and Metabolism at the Shanghai Tenth People’s Hospital. Anthropometric characteristics, metabolic parameters, and reproductive hormones were measured. Hyperuricemia was defined as serum uric acid (SUA) greater than 420 μmol/l. Dual-energy X-ray absorptiometry (DEXA) was used to measure body fat distribution.

**Results:**

The prevalence of hyperuricemia in patients with PCOS was 28.64%. PCOS patients with hyperuricemia are more obese and have a higher waist-to-hip ratio (WHR) and worse lipid metabolism than those without hyperuricemia. According to SUA quartiles, patients in the highest quartile had higher total testosterone (TT), body fat accumulation, and lower sex hormone-binding globulin (SHBG) than patients in the lowest quartile. SUA was correlated with percentage of total body fat, arm fat mass, leg fat mass, trunk fat mass, android/gynoid (A/G) ratio, and visceral adipose tissue (VAT) mass. After controlling possible confounders, logistic regression analysis found that only excessive VAT mass could significantly increase the risk of hyperuricemia in patients with PCOS.

**Conclusion:**

In patients with PCOS, a high level of VAT mass, but not other fat compartments, will exacerbate the risk of hyperuricemia. Attention should be paid to the role of excessive VAT in the occurrence and development of PCOS with hyperuricemia.

## Introduction

Polycystic ovary syndrome (PCOS) is a common endocrine and metabolic disease in women of normal reproductive age, which is characterized by clinical and/or biochemical signs of androgen excess, ovulatory dysfunction, and polycystic ovarian morphology (1). Simultaneously, patients with PCOS often present abdominal obesity, which is not only observed in obese PCOS women but also in a large percentage of overweight PCOS women and a minority of normal weight PCOS women (2). The interplay between PCOS and abdominal adiposity is considered as a vicious circle of androgen excess favoring abdominal fat, which in turn facilitates androgen excess by the direct effects of several autocrine, paracrine, and endocrine mediators or indirectly by the induction of insulin resistance and hyperinsulinemia (3).

Previous observational studies have shown that the serum uric acid (SUA) levels and the prevalence of hyperuricemia were significantly higher in patients with PCOS than in patients without PCOS (4–7). To our knowledge, elevated SUA levels may have a variety of pro-inflammatory, pro-oxidative, vasoconstrictive actions and are commonly linked to the metabolic syndrome (8), including hypertension, atherosclerosis, and adiposity (9). In some populations other than PCOS patients, such as healthy people, diabetic patients, and obese people, many scholars have conducted research on the relationship between uric acid or hyperuricemia and adiposity/body fat distribution (10–17). However, as far as we know, there are few epidemiological studies evaluating the relationship between uric acid or hyperuricemia and body fat distribution in patients with PCOS.

The aim of this study was to determine the association between SUA and body fat distribution determined by dual-energy X-ray absorptiometry (DEXA) in patients with PCOS and to explore which fat measurement had a close correlation with the prevalence of hyperuricemia. Paying attention to the relationship between serum uric acid and body fat distribution in patients with PCOS has a predictive effect on early intervention of hyperuricemia and correction of metabolic disorders.

## Patients and Methods

### Study Design and Patients

**Figure 1:**
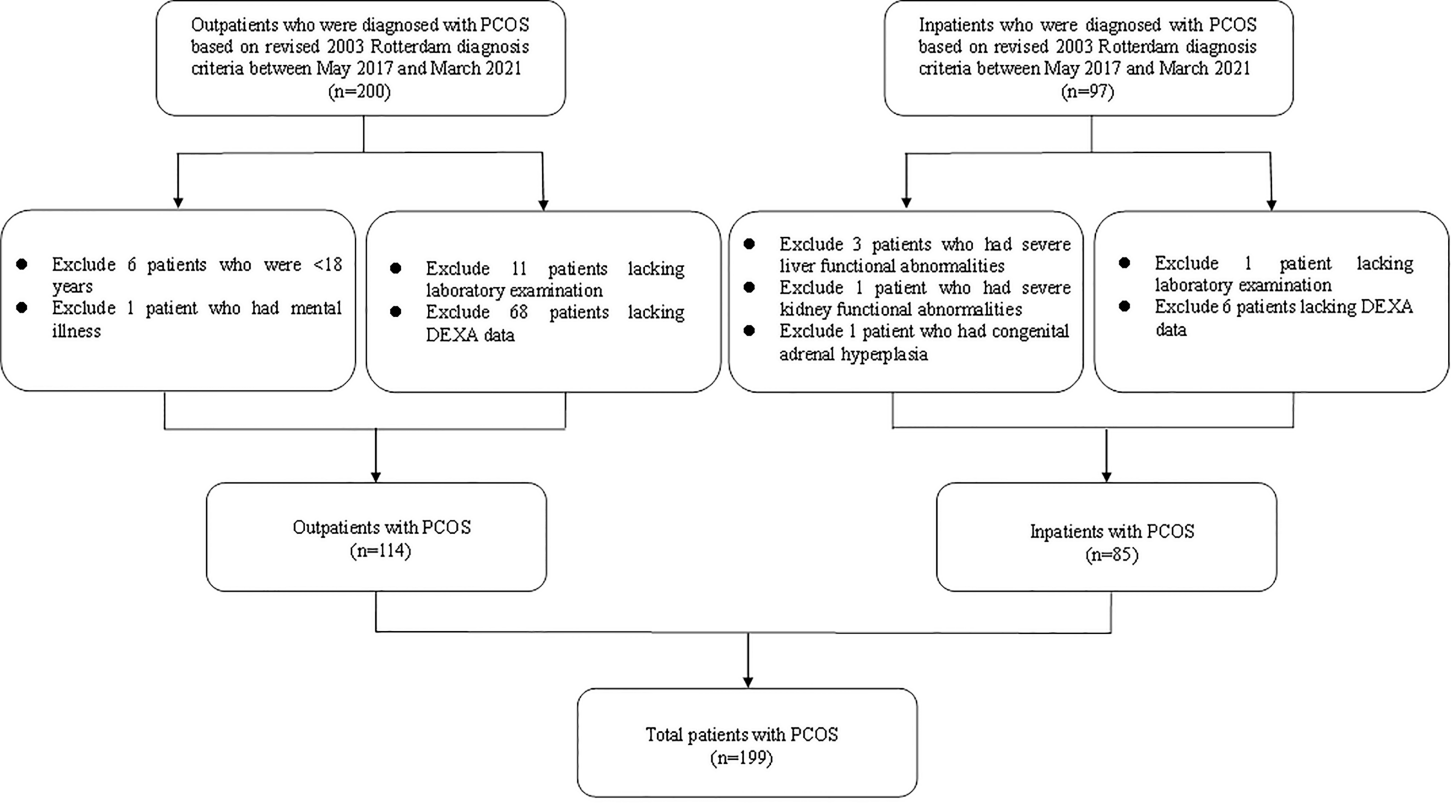
Flow diagram of the population included in the study.

A total 199 patients with PCOS who were admitted to the Department of Endocrinology and Metabolism at the Shanghai Tenth People’s Hospital between May 2017 and March 2021 were recruited in this cross-sectional study (**Figure 1**). PCOS was diagnosed based on the revised 2003 Rotterdam diagnosis criteria, in which at least two of the following three criteria should be involved: 1) oligo- or anovulation; 2) clinical (such as hirsutism and acne) and/or biochemical signs of hyperandrogenism; and 3) polycystic ovaries (presence of 12 or more follicles in each ovary measuring 2–9 mm in diameter, and/or increased ovarian volume >10 ml), and exclusion of other etiologies (congenital adrenal hyperplasia, androgen-secreting tumors, Cushing’s syndrome) (18). Exclusion criteria were 1) age <18 years or >45 years old; 2) severe liver and kidney functional abnormalities; 3) secondary obesity due to endocrine disorders; 4) mental illnesses that caused inability to provide informed consent; 5) malignant tumor; 6) the use of drugs that affect uric acid metabolism; and 7) incomplete laboratory examination or DEXA data. This cross-sectional study was approved by the ethics committee of Shanghai Tenth People’s Hospital. All individual participants enrolled were requested for written informed consent.

### Anthropometric Assessment and Laboratory Analysis

Trained endocrinology specialists measured and recorded the clinical data from all patients, including age, body weight, waist circumference (WC), hip circumference (HC), systolic blood pressure (SBP), and diastolic blood pressure (DBP). The body mass index (BMI) was calculated as body weight/(height × height) (kg/m^2^). The waist-to-hip ratio (WHR) was calculated as WC/HC. Signs of polycystic ovaries were detected *via* an ultrasound scan of pelvis. Blood samples were collected in the morning after at least 10 h of overnight fasting. Fasting plasma glucose (FPG), fasting insulin (FINS), fasting C peptide (F-CP), and glycosylated hemoglobin A1c (HbA1c) were determined by high-performance liquid chromatography. Postprandial plasma glucose (PPG) was measured by a 75-g oral glucose tolerance test, followed by examination at 2 h. Alanine aminotransferase (ALT), aspartate aminotransferase (AST), total cholesterol (TC), triglycerides (TG), low-density lipoprotein cholesterol (LDL-c), high-density lipoprotein cholesterol (HDL-c), creatinine (Cr), and SUA were measured by using enzymatic assays. Levels of reproductive hormones such as prolactin (PRL), free testosterone (FT), androstenedione (AD), and dehydroepiandrosterone sulfate (DHEAS) were measured. Luteinizing hormone (LH), follicle-stimulating hormone (FSH), total testosterone (TT), and sex hormone-binding globulin (SHBG) were measured using an electrochemiluminescence immunoassay (Roche Diagnostics GmbH, Cot., Sandhofer, Mannheim, Germany). Homeostatic model assessment of insulin resistance (HOMA-IR) was calculated using the formula [FPG (mmol/L) × FINS (mU/L)]/22.5 (19). Metabolic syndrome (MS) was defined according to the criteria of the Chinese Diabetes Society, and more than three of the following components must be included: 1) WC (as measure of central obesity or abdominal obesity) ≥ 90 cm (male) or ≥ 85 cm (female); 2) high serum glucose level: fasting plasma glucose (FPG) ≥ 6.1 mmol/l and (or) 2 hPPG ≥ 7.8 mmol/l and (or) have been diagnosed as diabetes and undergone treatment; 3) blood pressure (BP): systolic blood pressure ≥ 130 mmHg and/or diastolic pressure ≥ 85 mmHg and (or) have been diagnosed as hypertensive and undergone treatment; 4) fasting blood TG ≥ 1.7 mmol/l; and 5) fasting blood HDL-C < 1.04 mmol/l (20). Hyperuricemia was defined as SUA greater than 420 μmol/l (21).

### Body Fat Distribution Measurements

Body fat distribution in PCOS patients was assessed by dual-energy X-ray absorptiometry (DEXA, APEX 4.5.0.2, Hologic, USA). The fat mass was measured in the whole body and six different regions, including head, arms, legs, trunk, android, and gynoid regions. The android/gynoid ratio (A/G ratio) was calculated as percentage of android fat/percentage of gynoid fat. The visceral adipose tissue (VAT) mass, VAT volume, and VAT area were automatically computed using the DEXA software. Abdominal subcutaneous adipose tissue (SAT) mass = android fat mass - VAT mass (13). VAT/SAT ratio was calculated as VAT mass/abdominal SAT mass.

### Statistical Analysis

All statistical analysis was performed using SPSS version 25.0 (SPSS Inc.; Chicago, IL, USA). All normal distribution continuous variables were presented as mean ± standard deviation (SD). Non-normal distribution continuous data were expressed as median and interquartile range (25%, 75%). Comparison continuous data among three subgroups were analyzed using one-way ANOVA test, followed by pairwise comparisons using least significant difference (LSD). Comparison continuous data between two subgroups were performed using the independent sample t-test. Comparison categorical data between groups were analyzed using the chi-squared test. Correlation analysis was performed using Pearson correlation tests. Multiple logistic regression analysis was performed to explore the association between SUA and body fat distribution in patients with PCOS. We included the variable with p < 0.05 in univariate analysis and the possible confounders but excluded the similar variable to avoid the co-linearity determinations in logistic regression models. The two-tailed p value <0.05 was considered statistically significant.

## Results

### General Characteristics of the PCOS Participants Stratified by BMI

**Table 1** shows anthropometric, metabolic, hormonal characteristics and body fat distribution of the PCOS patients stratified by BMI. Mean SBP, FPG, HbA1c, and the rate of diabetes were much lower in normal-weight and overweight groups than in the group with obese. The mean HOMA-IR, PPG, FINS, F-CP, ALT, AST, SUA, HDL-c, SHBG, and the rate of metabolic syndrome were significantly different among the three groups. The rates of hyperuricemia in the normal-weight, overweight, and obese groups were 4.30%, 25.00%, and 40.20%, respectively (p < 0.001). There were no significant differences in mean FSH, TT, FT, AD, and DHEAS among the three groups. The characteristics of body fat distribution were significantly different in different BMI subgroups.

**Table 1:** Anthropometric, metabolic, and hormonal characteristics and body fat distribution of the patients with PCOS stratified by BMI.

Items	BMI < 25 kg/m^2^	25 ≤ BMI < 30 kg/m^2^	BMI ≥ 30 kg/m^2^	p values
	N = 47	N = 40	N = 112	
**Age (years)**	27.06 ± 3.96	28.25 ± 5.22	28.38 ± 5.13	0.294
**Weight (kg)**	56.02 ± 6.02	73.22 ± 6.80	102.77 ± 16.68	**<0.001*^a,b,c^***
**BMI (kg/m^2^)**	21.10 ± 2.11	27.35 ± 1.51	37.93 ± 5.21	**<0.001*^a,b,c^***
**WHR**	0.83 ± 0.06	0.91 ± 0.07	0.95 ± 0.06	**<0.001*^a,b,c^***
**SBP (mmHg)**	112.86 ± 11.13	119.36 ± 10.39	131.09 ± 13.87	**<0.001*^b,c^***
**DBP (mmHg)**	75.95 ± 9.99	81.77 ± 9.38	83.23 ± 11.08	**0.018*^b^***
**FPG (mmol/L)**	4.76 ± 0.46	5.10 ± 0.51	6.07 ± 1.95	**<0.001*^b,c^***
**PPG (mmol/L)**	6.03 ± 1.41	7.73 ± 2.38	9.56 ± 3.88	**<0.001*^a,b,c^***
**FINS (mU/L)**	8.42 ± 4.50	19.08 ± 9.11	34.26 ± 20.11	**<0.001*^a,b,c^***
**F-CP (mmol/L)**	1.85 ± 0.52	2.97 ± 1.23	4.49 ± 1.38	**<0.001*^a,b,c^***
**HbA1c (%)**	5.40 ± 0.27	5.56 ± 0.50	6.01 ± 1.28	**0.001*^b,c^***
**HOMA-IR**	1.83 ± 1.21	4.35 ± 2.15	9.39 ± 6.37	**<0.001*^a,b,c^***
**ALT (U/L)**	18.24 ± 14.11	38.81 ± 48.08	55.57 ± 42.37	**<0.001*^a,b,c^***
**AST (U/L)**	19.14 ± 7.49	26.44 ± 23.73	35.00 ± 28.72	**0.001*^b^***
**TC (mmol/L)**	4.75 ± 1.16	4.73 ± 0.83	4.58 ± 0.76	0.442
**TG (mmol/L)**	0.88 ± 0.37	1.62 ± 0.89	1.92 ± 1.39	**<0.001*^a,b^***
**LDL-c (mmol/L)**	2.76 ± 1.02	3.03 ± 0.76	2.79 ± 0.68	0.214
**HDL-c (mmol/L)**	1.64 ± 0.44	1.24 ± 0.21	1.05 ± 0.25	**<0.001*^a,b,c^***
**SUA (μmol/L)**	302.10 ± 63.73	375.25 ± 73.43	410.21 ± 82.92	**<0.001*^a,b,c^***
**Metabolic syndrome (%,n)**	2.10 (1)	25.00 (10)	60.70 (68)	**<0.001*^a,b,c^***
**Diabetes (%,n)**	2.10 (1)	12.50 (5)	39.30 (44)	**<0.001*^b,c^***
**Hyperuricemia (%,n)**	4.30 (2)	25.00 (10)	40.20 (45)	**<0.001*^a,b^***
**LH (IU/L)**	11.61 ± 6.97	11.79 ± 7.57	7.45 ± 4.01	**<0.001*^b,c^***
**FSH (IU/L)**	5.23 ± 1.96	5.18 ± 2.18	4.70 ± 1.70	0.176
**PRL (mIU/L)**	397.03 ± 216.21	367.52 ± 158.72	505.66 ± 266.82	**0.002*^b,c^***
**TT (nmol/Ll)**	1.64 ± 0.59	1.82 ± 0.96	1.71 ± 0.86	0.613
**FT (pg/ml)**	2.57 ± 1.26	2.37 ± 1.36	2.07 ± 1.30	0.404
**AD (ng/ml)**	4.52 ± 1.72	4.17 ± 1.61	4.47 ± 1.90	0.670
**DHEAS (μg/dl)**	265.97 ± 116.87	224.18 ± 120.36	227.74 ± 100.19	0.241
**SHBG (nmol/L)**	49.20 ± 26.31	36.93 ± 33.73	19.56 ± 12.06	**<0.001*^a,b,c^***
**Total body fat (%)**	35.95 ± 4.98	41.03 ± 3.46	46.70 ± 3.74	**<0.001*^a,b,c^***
**Total fat mass (g)**	20041.29 ± 4220.9	29656.39 ± 4368.24	46739.88 ± 9757.65	**<0.001*^a,b,c^***
**Android fat mass (g)**	1399.49 ± 427.35	2276.80 ± 448.58	4317.41 ± 1283.01	**<0.001*^a,b,c^***
**Gynoid fat mass (g)**	3631.83 ± 708.93	4574.08 ± 811.50	6660.791588.41	**<0.001*^a,b,c^***
**A/G ratio**	0.91 ± 0.13	1.07 ± 0.13	1.17 ± 0.14	**<0.001*^a,b,c^***
**VAT mass (g)**	325.15 ± 115.40	627.43 ± 168.07	1119.40 ± 305.63	**<0.001*^a,b,c^***
**Abdominal SAT mass (g)**	1074.34 ± 323.16	1649.38 ± 324.63	3196.64 ± 1064.64	**<0.001*^a,b,c^***
**VAT/SAT ratio**	0.30 ± 0.05	0.38 ± 0.08	0.36 ± 0.08	**<0.001*^a,b^***

The data are presented as the mean ± standard deviation.

^a^Significant difference between BMI < 25 kg/m^2^ group and 25 ≤ BMI < 30 kg/m^2^ group.

^b^Significant difference between BMI < 25 kg/m^2^ group and BMI ≥ 30 kg/m^2^ group.

^c^Significant difference between 25 ≤ BMI < 30 kg/m^2^ group and BMI ≥ 30 kg/m^2^ group.

The bold values indicate statistical significance.

BMI, body mass index; WHR, waist/hip ratio; SBP, systolic blood pressure; DBP, diastolic blood pressure; FPG, fasting plasma glucose; PPG, postprandial plasma glucose; FINS, fasting insulin; F-CP, fasting C peptide; HbA1c, glycosylated hemoglobin A1c; HOMA-IR, homeostasis model assessment of insulin resistance; ALT, alanine aminotransferase; AST, aspartate aminotransferase; TC, total cholesterol; TG, triglyceride; LDL-c, low-density lipoprotein cholesterol; HDL-c, high-density lipoprotein cholesterol; SUA, serum uric acid; LH, luteinizing hormone; FSH, follicle-stimulating hormone; PRL, pituitary prolactin; TT, total testosterone; FT, free testosterone; AD, androstenedione; DHEAS, dehydroepiandrosterone sulfate; SHBG, sex hormone-binding globulin; A/G ratio, android/gynoid ratio; VAT, visceral adipose tissue; SAT, subcutaneous adipose tissue.

### Anthropometric and Metabolic Characteristics of the PCOS Participants in the Non-Hyperuricemia Group and Hyperuricemia Group

The anthropometric and metabolic characteristics of the patients with PCOS stratified by presence of hyperuricemia are displayed in **Table 2**. Our study comprised 199 women with PCOS (mean age 28.04 ± 4.91 years). The prevalence of hyperuricemia was up to 28.64%. The group of PCOS patients with hyperuricemia had higher weight and BMI than the non-hyperuricemia group. After adjustment for BMI, mean WHR were higher in the group of PCOS patients with hyperuricemia than the group with non-hyperuricemia. The mean TC and LDL-c levels in the group of PCOS patients with hyperuricemia were higher than those of the non-hyperuricemia group. However, the mean SBP, DBP, FPG, PPG, FINS, F-CP, HbA1c, HOMA-IR, ALT, AST, TG, and HDL-c and Cr were comparable between the two groups.

**Table 2:** Anthropometric and metabolic characteristics of the patients with PCOS in total, non-hyperuricemia group and hyperuricemia group.

Items	Total	Non-hyperuricemia	Hyperuricemia	p values
	N = 199	N = 142 (71.36%)	N = 57 (28.64%)	
**Age (years)**	28.04 ± 4.91	28.00 ± 4.80	28.14 ± 5.20	0.856
**Weight (kg)**	85.79 ± 24.06	81.14 ± 24.17	97.38 ± 19.60	**<0.001**
**BMI (kg/m^2^)**	31.83 ± 8.31	30.26 ± 8.42	35.75 ± 6.62	**<0.001**
**WC (cm)**	100.56 ± 19.03	96.88 ± 19.55	109.99 ± 13.83	0.088*
**HC (cm)**	109.57 ± 14.35	107.11 ± 14.36	116.00 ± 12.31	0.814*
**WHR**	0.91 ± 0.08	0.90 ± 0.08	0.95 ± 0.07	0.040*
**SBP (mmHg)**	126.35 ± 14.79	123.73 ± 13.02	132.02 ± 16.83	0.076*
**DBP (mmHg)**	81.85 ± 10.91	80.86 ± 10.14	83.98 ± 12.27	0.322*
**FPG (mmol/L)**	5.57 ± 1.60	5.44 ± 1.58	5.88 ± 1.63	0.932*
**PPG (mmol/L)**	8.38 ± 3.51	8.12 ± 3.62	9.00 ± 3.19	0.864*
**FINS (mU/L)**	25.00 ± 19.18	21.48 ± 16.93	33.50 ± 21.68	0.075*
**F-CP (mmol/L)**	3.94 ± 1.60	3.63 ± 1.60	4.61 ± 1.40	0.093*
**HbA1c (%)**	5.78 ± 1.04	5.72 ± 1.01	5.92 ± 1.09	0.800*
**HOMA-IR**	6.55 ± 5.89	5.55 ± 5.40	8.95 ± 6.36	0.161*
**ALT (U/L)**	43.47 ± 41.80	36.97 ± 38.09	59.33 ± 46.35	0.063*
**AST (U/L)**	29.52 ± 25.10	26.43 ± 21.55	37.12 ± 31.15	0.193*
**TC (mmol/L)**	4.65 ± 0.88	4.57 ± 0.77	4.86 ± 1.09	0.013*
**TG (mmol/L)**	1.62 ± 1.21	1.46 ± 1.15	2.01 ± 1.26	0.068*
**LDL-c (mmol/L)**	2.83 ± 0.79	2.76 ± 0.68	3.01 ± 0.98	0.041*
**HDL-c (mmol/L)**	1.22 ± 0.39	1.28 ± 0.41	1.07 ± 0.26	0.061*
**Cr (μmol/L)**	58.44 ± 8.55	58.36 ± 8.31	58.64 ± 9.19	0.218*

The data are presented as the mean ± standard deviation.

*p-values represented the results after adjustment for BMI.

The bold values indicate statistical significance.

BMI, body mass index; WC, waist circumference; HC, hip circumference; WHR, waist/hip ratio; SBP, systolic blood pressure; DBP, diastolic blood pressure; FPG, fasting plasma glucose; PPG, postprandial plasma glucose; FINS, fasting insulin; F-CP, fasting C peptide; HbA1c, glycosylated hemoglobin A1c; HOMA-IR, homeostasis model assessment of insulin resistance; ALT, alanine aminotransferase; AST, aspartate aminotransferase; TC, total cholesterol; TG, triglyceride; LDL-c, low-density lipoprotein cholesterol; HDL-c, high-density lipoprotein cholesterol; Cr, creatinine.

### Reproductive Hormones in the PCOS Patients According to SUA Quartiles

The overall PCOS patients were split into four groups across the SUA quartiles. No significant differences were observed in the mean LH, FSH, PRL, FT, AD, and DHEAS based on SUA quartiles. Compared with the participants in the first quartile, those in the fourth quartile had an evidently higher TT level and lower SHBG level (**Table 3**).

**Table 3:** Reproductive hormones and body fat distribution characteristics of the patients with PCOS across quartiles of serum uric acid.

Items	Quartiles of SUA				p values for trend
	Q1	Q2	Q3	Q4	
**n**	50	51	50	48	–
**Median (25–75th percentiles), SUA**	275.75 (259.00,296.00)	348.00 (332.80,362.80)	405.00 (390.40,419.00)	469.95 (452.50,534.40)	–
**BMI (kg/m^2^)**	25.81 ± 8.50	31.76 ± 7.91	34.32 ± 6.36	35.59 ± 6.91	**<0.001**
**LH (IU/L)**	10.48 ± 6.91	9.29 ± 6.70	8.78 ± 5.26	8.64 ± 4.84	0.118
**FSH (IU/L)**	5.26 ± 2.20	4.92 ± 1.57	4.98 ± 1.95	4.53 ± 1.69	0.078
**PRL (mIU/L)**	435.84 ± 200.73	424.33 ± 288.31	549.00 ± 375.26	443.23 ± 214.41	0.506
**TT (nmol/L)**	1.52 ± 0.66	1.65 ± 0.86	1.74 ± 0.75	1.96 ± 0.97	**0.007**
**FT (pg/ml)**	2.32 ± 1.38	2.34 ± 1.35	2.80 ± 1.24	2.06 ± 1.07	0.817
**AD (ng/ml)**	4.45 ± 1.80	4.10 ± 1.69	4.31 ± 1.68	4.74 ± 1.72	0.518
**DHEAS (μg/dl)**	229.19 ± 109.30	253.42 ± 132.37	236.95 ± 93.53	258.24 ± 129.09	0.518
**SHBG (nmol/L)**	45.20 ± 20.22	35.49 ± 31.84	20.59 ± 13.97	29.33 ± 34.15	**0.007**
**Total body fat (%)**	38.06 ± 7.03	44.44 ± 4.99	44.71 ± 4.33	44.93 ± 4.50	**<0.001**
**Total fat mass (g)**	26834.13 ± 14893.97	37524.76 ± 13321.92	41291.92 ± 10420.79	42562.46 ± 11089.12	**<0.001**
**Head fat mass (g)**	1228.02 ± 471.07	1312.08 ± 266.77	1527.99 ± 613.48	1448.36 ± 371.93	**0.003**
**Arms fat mass (g)**	3491.80 ± 2180.33	4963.52 ± 2013.05	5765.38 ± 1692.88	6102.92 ± 2137.17	**<0.001**
**Legs fat mass (g)**	8905.88 ± 3680.59	11730.22 ± 3612.81	12987.75 ± 3921.87	12946.22 ± 3670.95	**<0.001**
**Trunk fat mass (g)**	13207.94 ± 8961.73	19539.48 ± 8494.41	21028.66 ± 5640.17	21677.71 ± 6678.53	**<0.001**
**Android fat mass (g)**	2160.98 ± 1540.64	3233.76 ± 1690.33	3727.54 ± 1464.01	3771.88 ± 1283.55	**<0.001**
**Gynoid fat mass (g)**	4353.74 ± 1718.66	5555.67 ± 1753.68	6152.82 ± 1844.41	6061.46 ± 1534.88	**<0.001**
**A/G ratio**	0.97 ± 0.19	1.11 ± 0.17	1.13 ± 0.12	1.14 ± 0.14	**<0.001**
**VAT mass (g)**	521.84 ± 368.10	816.00 ± 378.89	967.86 ± 385.44	1024.11 ± 369.93	**<0.001**
**VAT volume (cm^3^)**	564.10 ± 397.95	880.06 ± 412.76	1046.36 ± 416.66	1106.22 ± 399.77	**<0.001**
**VAT area (cm^2^)**	108.20 ± 76.37	169.27 ± 78.52	200.71 ± 79.90	212.45 ± 76.68	**<0.001**
**Abdominal SAT mass (g)**	1639.14 ± 1187.28	2405.94 ± 1371.25	2759.68 ± 1127.04	2710.11 ± 970.09	**<0.001**
**VAT/SAT ratio**	0.32 ± 0.06	0.35 ± 0.08	0.36 ± 0.08	0.38 ± 0.07	**<0.001**

The data are presented as the mean ± standard deviation and median (interquartile range). The bold values indicate statistical significance.

SUA, serum uric acid; BMI, body mass index; LH, luteinizing hormone; FSH, follicle-stimulating hormone; PRL, pituitary prolactin; TT, total testosterone; FT, free testosterone; AD, androstenedione; DHEAS, dehydroepiandrosterone sulfate; SHBG, sex hormone-binding globulin; A/G ratio, android/gynoid ratio; VAT, visceral adipose tissue; SAT, subcutaneous adipose tissue.

### Body Fat Distribution in the PCOS Patients According to SUA Quartiles

The characteristics of body fat distribution in the PCOS patients were presented according to SUA quartiles in **Table 3**. The percentage of total body fat and total fat mass increased across the SUA quartiles in total PCOS patients. Participants in the fourth quartile of SUA had a significantly higher head fat mass, arm fat mass, leg fat mass, trunk fat mass, android fat mass, gynoid fat mass, abdominal SAT mass, A/G ratio, and VAT/SAT ratio than the patients in the first quartile of SUA. Meanwhile, the trends in the increase in the VAT mass, VAT volume, and VAT area associated with SUA level were statistically significant (p for trend <0.001).

### Association Between Body Fat Distribution and SUA or Hyperuricemia

**Figure 2:**
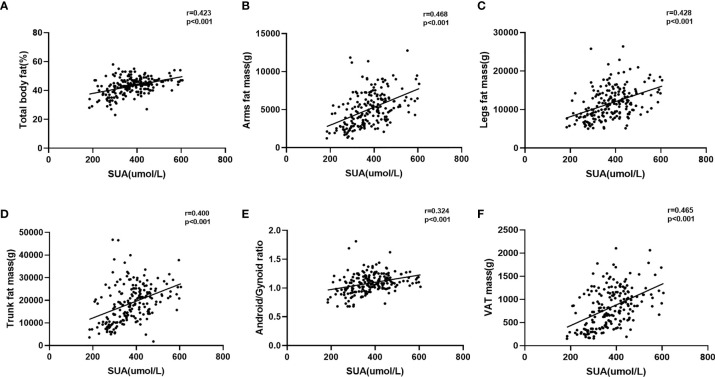
Correlation between serum uric acid and body fat distribution. **(A)** Correlation between serum uric acid and total body fat%; **(B)** correlation between serum uric acid and arm fat mass; **(C)** correlation between serum uric acid and leg fat mass; **(D)** correlation between serum uric acid and trunk fat mass; **(E)** correlation between serum uric acid and android/gynoid ratio; **(F)** correlation between serum uric acid and VAT mass.

As shown in **Figure 2**, the SUA level was significantly positively correlated with percentage of total body fat, arm fat mass, leg fat mass, trunk fat mass, A/G ratio, and VAT mass (r = 0.423, 0.468, 0.428, 0.400, 0.324, 0.465, respectively; p < 0.001 for all). In order to determine the influence of different degrees of characteristics related to body fat distribution on hyperuricemia, we dichotomized the PCOS patients according to characteristics related to body fat distribution and performed multiple logistic regression models to evaluate the risk of hyperuricemia between the two groups (**Table 4**). After adjustment for age (Model 1), we found that the higher levels of percentage of total body fat, trunk fat mass, android fat mass, gynoid fat mass, VAT mass, and abdominal SAT mass will increase the risk of hyperuricemia in PCOS patients. With further adjustment for confounders in multiple logistic regression models (Model 2–4), only higher level of VAT mass was significantly associated with hyperuricemia (p < 0.05 in all three models).

**Table 4:** Logistic regression analysis of different degrees of body fat distribution characteristics and hyperuricemia.

	Total body fat (%)					Trunk fat mass(g)					Android fat mass (g)			
	<43.70	≥43.70			p values	<18,575.70	≥18,575.70			p values	<2,975.00	≥2,975.00	p values	
	Reference	OR (95% CI)				Reference	OR (95% CI)				Reference	OR (95% CI)		
**Model 1**	1	2.733 (1.435,5.207)			**0.002**	1	3.714 (1.903,7.249)			**<0.001**	1	4.191 (2.123,8.275)	**<0.001**	
**Model 2**	1	1.581 (0.553,4.516)			0.392	1	2.835 (0.749,10.721)			0.125	1	2.676 (0.738,9.710)	0.134	
**Model 3**	1	1.432 (0.435,4.719)			0.555	1	1.764 (0.399,7.794)			0.454	1	2.025 (0.477,8.597)	0.339	
**Model 4**	1	1.455 (0.423,5.006)			0.552	1	2.450 (0.516,11.638)			0.260	1	2.740 (0.591,12.708)	0.198	
	**Gynoid fat mass (g)**					**VAT mass (g)**					**Abdominal SAT mass (g)**			
	**<5,204.00**	**≥5,204.00**			**p values**	**<819.50**	**≥819.50**			**p values**	**<2,149.00**	**≥2,149.00**	**p values**	
	**Reference**	**OR (95% CI)**				**Reference**	**OR (95% CI)**				**Reference**	**OR (95% CI)**		
**Model 1**	1		3.327 (1.721,6.431)	**<0.001**		1		5.176 (2.510,10.675)	**<0.001**		1	3.823 (1.931,7.568)		**<0.001**
**Model 2**	1		1.232 (0.430,3.535)	0.697		1		6.409(1.670,24.589)	**0.007**		1	2.023(0.545,7.510)		0.293
**Model 3**	1		1.070 (0.343,3.345)	0.907		1		5.292 (1.228,22.809)	**0.025**		1	1.657 (0.387,7.101)		0.496
**Model 4**	1		1.452(0.406,5.198)	0.566		1		7.258(1.483,35.529)	**0.014**		1	1.779(0.395,8.014)		0.453

OR, odds ratio; CI, confidence interval. Model 1: adjusted for age; Model 2: adjusted for age, BMI, hypertension; Model 3: adjusted for age, BMI, hypertension, HbA1c, HOMA-IR, ALT, TG; Model 4: adjusted for age, BMI, hypertension, HbA1c, HOMA-IR, ALT, TG, TT. The bold values indicate statistical significance.

## Discussion

Multiple studies have shown that the SUA levels and the prevalence of hyperuricemia were significantly higher in patients with PCOS than in patients without PCOS (4–7). Simultaneously, patients with PCOS often present abdominal obesity, which is associated with hyperinsulinemia and elevated androgen. Considering hyperuricemia could exacerbate insulin resistance and metabolic dysfunction (22), it is interesting to explore the association between uric acid and body fat distribution in patients with PCOS. To the best of our knowledge, the present study is the first study to investigate the relationship between uric acid and body fat distribution in patients with PCOS.

In our study, 28.64% of patients with PCOS had hyperuricemia, which is in accordance with previous studies showing that the prevalence of hyperuricemia in the PCOS population varied from 25.48% to 26.29%, almost threefold higher than that of women in the general population (4, 23). Consistent with previous studies (4, 24), our data have shown that SUA levels were positively associated with elevated total testosterone in women with PCOS. It was reported that testosterone could increase SUA levels by inducing the hepatic metabolism of purine nucleotides (25) or by upregulating the expression of Smct1 among the urate re-absorptive transport system (26). Moreover, the administration of anti-androgenic contraceptives ameliorated androgen excess, which was in parallel to the reduction of SUA levels in obese PCOS patients (27). A previous study has shown that SUA concentrations were independently linked with free testosterone in young healthy women (28). However, no linear trend of SUA levels increasing with elevated free testosterone levels was observed in our study. The discrepancies in these findings might be attributed to the differences in study populations, and sample size.

Our present results found that the PCOS patients with hyperuricemia were prone to be obese, which was consistent with a previous study that a high SUA level had a significant association with the increased risk of obesity (29). After adjusting for BMI, the group of PCOS patients with hyperuricemia had significantly higher WHR, TC, and LDL-c than the non-hyperuricemia group. Notably, our data indicated that the SHBG level in the highest SUA quartile was lower than that in the lowest SUA quartile. These results were in accordance with a cross-sectional study showing that SHBG was inversely correlated with SUA levels in premenopausal obese women (30). The underlying mechanism of this decrease in SHBG levels might be associated with inactivating AMPK in the hepatocytes brought about by elevated SUA concentration (31, 32). Taken together, our results were generally in line with previous studies showing a positive relationship for SUA with worse metabolic profiles in the PCOS population (33–35). It was reported that high levels of SUA could induce oxidative stress, affect lipid synthesis and lipid oxidation distortion, and aggravate systemic sterile inflammation, endothelial injury, and promote thrombosis, which could lead to the development of future cardiovascular morbidity in women with PCOS (36).

Increasing evidence has shown that patients with PCOS often present some degree of abdominal obesity, which has strong independent associations with both insulin resistance and hyperandrogenism (37–39). Given that hyperuricemia could also exacerbate the development of hyperandrogenism and insulin resistance in the PCOS population, it is important to investigate whether body fat distribution has a close relationship with SUA in those patients. For all participants, an increasing trend for mean levels of fat contents was found across the SUA levels. In line with previous studies (15, 40), higher contents of percentage of total body fat, trunk fat mass, android fat mass, gynoid fat mass, VAT mass, and abdominal SAT mass showed a significantly positive association with the risk of hyperuricemia. However, after adjusting for all confounder factors, only VAT mass was significantly correlated with the high risk of hyperuricemia in PCOS patients. These findings were consistent with previous studies, which have demonstrated that visceral adiposity, but not other fat compartments, was related to SUA levels in the general population (10–12). Similarly, a study reported that the visceral adiposity index was significantly positively correlated with uric acid in patients with PCOS (41). Therefore, the absence of any association between other fat compartments and the risk of hyperuricemia suggest that VAT may have a greater adverse influence on uric acid metabolism than any other fat compartments (17). Compared with SAT and other fat deposits, VAT adipocytes are more metabolically active with a greater capacity to secrete free fatty acids, adipocytokines, and other vasoactive substances, which may influence the risk of developing impaired glucose tolerance, hyperinsulinemia, and hypertriglyceridemia (42, 43). Further studies are needed to clarify the potential pathogenic effect of visceral adiposity on uric acid metabolism.

There were several limitations in our study. First, the sample size of the study was relatively small and the majority of the patients with PCOS were obese, which might introduce a selective bias. Second, we only did a cross-sectional study and could not clarify the causality and mechanisms underlying the relationship of adipose tissue to hyperuricemia. Further research on larger and longitudinal cohort studies and specific cell and animal experiments designed for the mechanism underlying the association between body fat distribution and hyperuricemia are warranted to clarify our consistent findings. In addition to limitations, a major advantage is that this study filled current gaps in literature by evaluating the effect of VAT fat accumulation on SUA level in the PCOS population, which is meaningful to ameliorate metabolic abnormalities and other dysfunction in the development of PCOS.

## Conclusion

PCOS patients with hyperuricemia were more obese and had higher WHR and worse lipid metabolism than those without hyperuricemia. VAT, but not other fat compartments, was significantly positively correlated with the high risk of hyperuricemia in the PCOS patients. Attention should be paid to the role of excessive VAT in the occurrence and development of PCOS with hyperuricemia.

## Data Availability Statement

The raw data supporting the conclusions of this article will be made available by the authors, without undue reservation.

## Ethics Statement

The studies involving human participants were reviewed and approved by the Institutional Human Subjects Review Board of Shanghai Tenth People’s Hospital. The patients/participants provided their written informed consent to participate in this study.

## Author Contributions

MZ and XS: designed and wrote. YZ, MC, and DD: performed and collected. ZL, HS, RC, HF, XG, and QZ: participated in recruiting the patients. SQ: edited. All authors contributed to the article and approved the submitted version.

## Funding

This study is supported by grants from the National Key R&D Program of China (No. 2018YFC1314100) and the National Nature Science Foundation (No. 81601269).

## Conflict of Interest

The authors declare that the research was conducted in the absence of any commercial or financial relationships that could be construed as a potential conflict of interest.

## Publisher’s Note

All claims expressed in this article are solely those of the authors and do not necessarily represent those of their affiliated organizations, or those of the publisher, the editors and the reviewers. Any product that may be evaluated in this article, or claim that may be made by its manufacturer, is not guaranteed or endorsed by the publisher.
